# Health-Related Quality of Life in Adrenocortical Carcinoma

**DOI:** 10.3390/cancers11101500

**Published:** 2019-10-08

**Authors:** Rebecca V. Steenaard, Laura A. Michon, Harm R. Haak

**Affiliations:** 1 Department of Internal Medicine, Máxima MC, 5631 Eindhoven/Veldhoven, The Netherlands; 2 CAPHRI School for Public Health and Primary Care, Ageing and Long-Term Care, Maastricht University, 6200 Maastricht, The Netherlands; 3 Department of Internal Medicine, Division of General Internal Medicine, Maastricht University Medical Centre+, 6229 Maastricht, The Netherlands

**Keywords:** adrenocortical carcinoma, health-related quality of life, adrenalectomy, mitotane, chemotherapy

## Abstract

Insight into the health-related quality of life (HRQoL) impact of adrenocortical carcinoma (ACC) is important. The disease and its treatment options potentially have an impact on HRQoL. For patients with limited survival, HRQoL research is of utmost importance. We will therefore provide an overview of HRQoL studies in patients with ACC. We found six studies that measured HRQoL in 323 patients with ACC (3 cross-sectional, 1 cohort, 2 trials), all indicating a reduced HRQoL compared to the general population. The FIRMACT trial found that HRQoL of patients with ACC was reduced compared to the general population, and that chemotherapy-mitotane further reduced HRQoL even though survival improved. Clinical aspects of the disease, including cortisol and aldosterone production and adrenal insufficiency have shown great impact on HRQoL in benign disease, even after the recovery of hormonal status. However, the impact of malignant adrenal disease and treatment options on HRQoL including adrenalectomy, radiotherapy, mitotane therapy, and chemotherapy have not been sufficiently studied in patients with ACC. Although the number of HRQoL studies in patients with ACC is limited, the existing literature does indicate that ACC has a large impact on patients’ HRQoL, with disease specific aspects. Further HRQoL research in patients with ACC is essential to improve patient-centered care, preferably by using an ACC-specific HRQoL questionnaire.

## 1. Introduction

Adrenocortical carcinoma (ACC) is a rare cancer with an incidence between 0.7 and 2.0 per million persons per year [1]. The overall median survival ranges from 5 to 159 months, and is strongly dependent on the stage of disease. ACC can present as a functional or nonfunctional tumor, with the latter often being an additional finding on imaging done for other purposes (incidentaloma finding). Treatment for ACC depends on the stage of disease [2,3]. The optimal treatment for solitary tumors is surgical resection. In cases with a high risk of recurrence (stage III, R1 or Rx resection, Ki67 > 10%), surgery is followed by at least 2 years of adjuvant mitotane monotherapy. For metastatic disease or irresectable tumors, the treatment consists of mitotane monotherapy, radiotherapy, or chemotherapy with or without the addition of mitotane.

Symptoms and treatment options of ACC can have a great impact on patients’ health-related quality of life (HRQoL). For example, both mitotane and chemotherapy are associated with high toxicity. Interviews with patients with ACC illustrate the importance of healthcare professionals having knowledge of the issues that patients face [4]. HRQoL questionnaires can help improve communication about these issues [5]. More knowledge of HRQoL issues using a HRQoL questionnaire can further assist in the process of shared-decision making and provide direction for further research [6,7].

HRQoL focuses on the impact of health on a person’s ability to live a fulfilling life. HRQoL questionnaires assess physical, mental, and social health influenced by disease and its treatments, and can be general or disease-specific [8]. General HRQoL questionnaires are useful for comparing HRQoL issues to other diseases, but they might result in an underreporting of HRQoL issues. For example, ACC symptoms include general cancer symptoms such as fatigue and weight-loss, but also more specific symptoms such as an increased abdominal size or virilization. A disease specific HRQoL questionnaire could help to better differentiate between issues experienced by different patient groups, at different stages of ACC, and during different treatment options. The PROQOLID database is a searchable clinical outcomes measurement database. It contains all existing validated questionnaires and clinical measurement tools for use in research and clinical practice. At the moment, this database does not include a disease specific model that is developed specifically for use in patients with ACC.

As recommended by the recent European Society for Endocrinology (ESE) guideline for ACC management, more knowledge of HRQoL in patients with ACC is necessary to improve patient-centered care [2]. This knowledge can firstly be used to aid the shared decision making process [9]. Current treatment decisions are mostly based on recurrence risk, expected treatment effect, and potential toxicity. However, the potential effect of a treatment on HRQoL is just as important for patients when deciding on a treatment. Secondly, healthcare professionals can use the information to improve patient doctor communication and improve patient satisfaction with care [5]. Knowledge of HRQoL can also be used to improve treatment guidance [6,7]. When we know which HRQoL issues occur during a specific stage of the treatment, steps can be taken to prevent, detect, and treat these issues. Finally, HRQoL knowledge can be helpful for implementation of quality improvement in clinical care [10].

In this systematic review, we will therefore provide an overview of the current knowledge of HRQoL in patients with ACC. A systematic search for papers in Medline, Cochrane library, Google Scholar, and Embase published in English until July 2019 resulted in a total of six studies that measured HRQoL in 323 patients with ACC, summarized in Table 1. We will present all aspects and treatments of ACC, its potential influence on HRQoL, and the studies measuring HRQoL in patients with ACC.

## 2. Results and Discussion

### 2.1. Clinical Presentation

#### 2.1.1. Functional Tumors

ACC presents with autonomous adrenal hormone production in 50–60% of the cases, most commonly with cortisol production or a combination of cortisol and androgen production [18]. Symptoms of cortisol-producing ACCs are often not comparable to full blown Cushing’s syndrome, but mostly include muscle weakness, hypokalemia, wasting, and constitutional symptoms [2,19]. Patients with excess cortisol levels experience a reduced HRQoL as recently described by the review of Ho and Druce [20]. While treatment is effective in improving HRQoL, patients with long-term remission still report a persistent effect on their HRQoL. This was mostly due to psychological impairments such as an increased risk of anxiety and depressive symptoms [21], negative illness perception, and impaired cognitive functioning with measurable changes in brain morphology due to hypercortisolism [22,23]. Furthermore, higher cortisol levels are associated with lower bone mineral density and higher fracture rates, especially in patients with ACC [24].

There are currently two HRQoL instruments in the questionnaire database PROQOLID which are developed specifically for use in patients with Cushing’s syndrome, namely the CushingQoL and Tuebingen CD-25 [25,26]. Since these questionnaires have not been developed especially for cancer patients, they are not fully applicable for use in patients with cortisol-producing ACCs. In our systematic review, we found one study that included three patients with cortisol-producing ACC (Table 1) [15]. This study reported a reduced HRQoL compared to the general population, even after biochemical remission using mifepristone. Further analysis of the same data showed that all domains of HRQoL were altered except for body pain [16]. However, the results of the patients with ACC were not provided separately in both studies, therefore no conclusions on the effect of cortisol-producing ACCs on HRQoL can be made based on these two studies.

ACCs presenting with aldosterone production are rare. Patients with hyperaldosteronism usually present with hypertension and hypokalemia or with cardiovascular events including atrial fibrillation [27]. Hyperaldosteronism is associated with reduced HRQoL in primary aldosteronism [20]. Patients can further experience an increased risk of anxiety and depression [28]. However, these studies did not include patients with ACC. A new questionnaire to measure HRQoL in patients with primary aldosteronism is currently being developed, the PA-QOL [29]. All patients used in the development phase were diagnosed with primary aldosteronism and no patients with malignant aldosterone producing tumors were invited to participate. Therefore, the questionnaire might not be fully applicable in patients with ACC. In our search, we found no studies on HRQoL including patients with aldosterone producing ACCs.

While ACCs producing both cortisol and androgens occur regularly, pure androgen- or estrogen-producing ACCs are rare. Female patients with an androgen-producing ACC present with hirsutism, menstrual irregularity, and virilization including clitoromegaly, male pattern baldness, deep voice, and acne. Male patients with an estrogen-producing ACC can experience gynecomastia, hypogonadism, and diminished libido [30]. In our search, we have found no studies that measured the HRQoL in patients with androgen- or estrogen-producing ACCs.

#### 2.1.2. Nonfunctional Tumors

Nonfunctional tumors most often present with abdominal pain, weight-loss, and nonspecific gastrointestinal symptoms such as nausea and anorexia [18,31]. More severe cases can present with invasive growth in internal organs including the adjacent kidney and vena cava. Metastases usually include lymph nodes, the lungs, and the liver. We found one study on HRQoL in gastro-intestinal endocrine tumors that included nine patients with a nonfunctional ACC (Table 1) [12]. Unfortunately, the HRQoL measurements were not provided. The study only described that patients who were satisfied with care had higher HRQoL and lower anxiety scores. Therefore, no conclusions regarding HRQoL in patients with nonfunctional ACC can be drawn from this study.

#### 2.1.3. Cancer Surveillance

ACC can be discovered accidentally on imaging done for other purposes or during incidentaloma follow-up. Follow-up for incidentaloma can have an impact on overall HRQoL and anxiety, due to patients’ concerns about possible malignancy of the tumor [32,33]. Similarly, it can be hypothesized that follow-up for recurrence after initial treatment of the tumor can have the same negative impact on HRQoL. Patients with ACC are seen by their healthcare professional every three months during treatment and for the first two years after treatment, and every three to six months for three more years. When no recurrence occurs, these check-ups are recommended to be continued with an adapted surveillance protocol [2]. This follow-up visit includes routine physical and laboratory checkups, and imaging of the thorax, abdomen, and pelvis to search for local disease recurrence or distant metastases. We found no studies on the potential HRQoL impact of this surveillance protocol.

During follow-up after ACC treatment, healthcare professionals also need to take into account that the impact of cancer on HRQoL does not end when treatment has been completed. It has been demonstrated that long-term cancer survivors still experience an impact on their HRQoL [34]. This impact is no longer caused by the acute symptoms of the disease and the adverse effects of treatment. Instead, patients suffer from other issues, such as changes in body image, health behavior, fatigue, negative health outlook, and financial insecurity. These effects on HRQoL have been studied in other types of cancer, but we have found no studies that included patients with ACC.

### 2.2. Surgery

Open adrenalectomy is the standard treatment for localized ACC or locally advanced ACC [2,3]. En bloc resection of the periadrenal fat, locoregional lymph nodes, and adjacent organs is recommended in case of suspected invasion. Complications of open adrenalectomy include injury to adjacent organs, bleeding, thrombosis, pneumothorax, and wound infections. Adrenalectomy for malignant tumors and hormonally active tumors has a higher complication rate, and is associated with a worse long-term outcome than adrenalectomy for benign adrenal tumors [35,36,37]. Two recent reviews showed that laparoscopic adrenalectomy for ACC has similar outcomes in tumor control but faster recovery time and higher patient satisfaction [38,39]. It is therefore concluded that a laparoscopic approach can be safe in selected patients with a tumor with a diameter less than six centimeters without evidence of local invasion, if the surgeon has sufficient experience [2].

We found three studies on HRQoL after adrenalectomy for benign and malignant adrenal tumors (Table 1) [11,13,17]. These three studies included seven patients with ACC, of whom four were alive during HRQoL measurement. However, none of the HRQoL measures were reported separately for the patients with ACC. One study showed that patients with a benign or malignant nonfunctional adrenal tumor experienced a slight decrease in HRQoL directly after surgery, mostly due to pain and limited physical activity [17]. HRQoL fully recovered in the weeks following surgery. This is in line with the findings of a study on adrenalectomy including only benign adrenal tumors [40].

Studies on benign and malignant cortisol-producing adrenal tumors showed an improvement of HRQoL after surgery due to recovery of hormonal status [11,13,41]. However, both studies used a single-question method to measure HRQoL. One used a visual analogue score (ranged from zero to ten) to indicate HRQoL, and the other used a change question (has your HRQoL been improved, yes or no). Furthermore, we hypothesize that these results might not be directly generalizable to patients with ACC, since the presentation in patients with ACC is often different from patients with benign causes of Cushing’s syndrome. Even though studies report no differences in HRQoL between different etiologies of Cushing’s syndrome (pituitary, adrenal or ectopic origin), these analysis did not include ACC as an etiology [13,42,43].

### 2.3. Radiotherapy

Radiotherapy has a smaller role in the treatment of primary ACC. Routine adjuvant radiotherapy after complete resection of small tumors is not recommended [2,3]. However, a recent systematic review did find a positive effect on local control [44]. Radiotherapy can play a role in improving local control of irresectable tumors or symptomatic metastasis [45,46], despite having no effect on overall survival [47]. Radiotherapy for ACC causes mostly mild acute toxicity such as nausea and fatigue, and only limited late toxicity [44,48]. Furthermore, radiotherapy provides pain relief, especially for vertebral and bone metastases, and can therefore have a potential positive effect on HRQoL. We found no studies on the HRQoL of patients who received radiotherapy for ACC, therefore there is no evidence yet supporting this hypothesis.

### 2.4. Mitotane Monotherapy

Mitotane therapy is used as adjuvant therapy for two years after surgery in patients without a macroscopic residual tumor and a perceived high risk of recurrence (stage III, R1 or Rx resection, Ki67 > 10%). For patients with an irresectable tumor or metastases, mitotane can be used in a palliative setting as monotherapy, or combined with chemotherapy [2,3]. Mitotane dosage is dependent on the performance status of the patient and the tolerability of the drug. The dosage is increased until a blood concentration between 14 mg/L and 20 mg/L is reached. Above a concentration of 20 mg/L the risks of adverse effects are highly increased.

Mitotane therapy has a high prevalence of adverse effects ranging from limited to severe. Table 2 provides an overview of adverse effects adapted from the recent ESE guidelines on the management of ACC [2]. Almost all patients experience gastro-intestinal adverse effects such as nausea and diarrhea. Most patients have laboratory disturbances including an increase in hepatic enzymes and hypercholesterolemia. Therefore, the use of supportive medication such as anti-emetics, antidiarrhea, and lipid lowering medication is recommended. All these adverse effects and the effects of supportive medication can potentially have an impact on patients’ HRQoL.

At higher serum concentrations, mitotane can cause neuropsychological adverse effects including vertigo, ataxia, impaired speech, and lethargy. More severe neuropsychological effects also occur, including cognitive problems such as impaired concentration and memory problems. There have even been reports of clinical depression with the need for antidepressants. As described in two papers in the early 1990s, these neuropsychological effects appear to be partly to completely reversible after cessation of mitotane therapy [49,50]. However, since these two papers these effects have not been studied in further research efforts.

In our search, we found no studies on HRQoL in patients who receive mitotane monotherapy. The only study that used a HRQoL questionnaire in mitotane users is the FIRMACT study (First International Randomized trial in locally advanced and Metastatic Adrenocortical carcinoma Treatment), which studies mitotane–chemotherapy combination therapy (Table 1) [14]. As it is difficult to interpret the results for mitotane therapy alone, we will describe the results in the chemotherapy section.

#### Adrenocortical Insufficiency

All patients on mitotane therapy develop adrenocortical insufficiency and require glucocorticoid supplementation, mostly in the form of hydrocortisone divided in one to three daily doses [2]. The supplementation of hydrocortisone in patients with ACC is different from the other forms of adrenal insufficiency due to CYP450 induction caused by mitotane. This results in high metabolism of several drugs, especially steroid based drugs such as hydrocortisone. Glucocorticoid supplementation in patients with ACC is therefore guided by clinical parameters only. This results, more often than in the other forms of adrenal insufficiency, in under- and overtreatment, leading to subsequent complaints affecting HRQoL.

Patients with adrenocortical insufficiency experience a reduced HRQoL, increased anxiety, and increased fatigue [20]. This HRQoL impact persists during adequate glucocorticoid supplementation. It is hypothesized that this persistent effect on HRQoL is due to the lack of a physiological cortisol rhythm during the day. Efforts to better simulate this rhythm with dual release preparations and three times per day dosages have not yet resulted in improved HRQoL [20]. Studies into the effects of additional mineralocorticoid and androgen replacement on HRQoL in patients with adrenocortical insufficiency have contradictory results [20]. The decision to add fludrocortisone or testosterone supplementation should therefore be based on clinical judgement and laboratory disturbances [2].

There is one disease-specific HRQoL questionnaire for use in patients with adrenocortical insufficiency, the AddiQoL [51]. This questionnaire was developed using patients with both primary and secondary causes of adrenal insufficiency, however, no patients with ACC were included. Therefore, the questionnaire might not be fully applicable for use in patients with ACC. For example, the impact of adrenal insufficiency on HRQoL in patients with mitotane therapy is unknown. Many of the symptoms of adrenocortical insufficiency overlap with the adverse effects of mitotane therapy, which include fatigue, anorexia, and myalgia (Table 2) [52]. It might therefore be difficult to distinguish the impact of adrenocortical insufficiency on HRQoL from the impact of mitotane therapy. We have found no HRQoL studies on adrenocortical insufficiency in our search that include patients with ACC or patients on mitotane therapy.

### 2.5. Chemotherapy

Chemotherapy is indicated for rapidly progressive or extensive metastatic disease, and for progression during mitotane monotherapy [2,3]. The first-line chemotherapy consists of etoposide, doxorubicin, and cisplatin (EDP), with or without the addition of mitotane. As a second-line treatment, streptozotocine–mitotane or gemcitabine–capecitabine can be considered [53,54]. Participation in clinical trials after failure of EDP chemotherapy can also be considered [2]. Other chemotherapeutic regimens and immunotherapy have shown little effectiveness thus far [18,46].

Patients on chemotherapy–mitotane combination therapy experience the same adverse effects as patients on mitotane monotherapy listed in Table 2 [2]. Additional adverse effects that are more specific to chemotherapy include bone-marrow toxicity, cardiovascular or thromboembolic events, loss of hair, and respiratory events [14,55]. These adverse effects can have a significant impact on HRQoL of patients undergoing chemotherapy [56].

The FIRMACT trial measured HRQoL in patients with ACC on chemotherapy–mitotane combination therapy and is the largest study on HRQoL in patients with ACC thus far (Table 1) [14]. The study compared the effectiveness of EDP–mitotane to streptozotocine–mitotane in 304 patients with ACC in a palliative setting. They used the general cancer HRQoL questionnaire developed by the European Organization for the Research and Treatment of Cancer (EORTC QLQ-C30) to measure HRQoL before and during treatment. They discovered that the EDP–mitotane regime had a better progression-free and overall survival, while HRQoL did not differ between the groups. They found that patients on both regimens had a reduced HRQoL before the start of the treatment (EORTC QLQ-C30 median score 58.3/100, *n* = 204). In comparison, the median score in the general population was 75.0/100 and in patients with metastatic colon cancer 66.7/100 [57]. HRQoL was even further affected during treatment (50.0/100, *n* = 129). However, this reduction was not significant due to the limited number of patients still alive during follow-up combined with a response rate of approximately 66%.

A limitation of the FIRMACT study is that they only used a general cancer HRQoL questionnaire. General HRQoL questionnaires do not cover the full range of specific ACC-related problems, which might result in underreporting of HRQoL issues. Furthermore, no data was provided on the different subscales of the EORTC-QLQ-C30. We therefore have no information on which domains of HRQoL are affected in patients with ACC on chemotherapy.

## 3. Materials and Methods

In this systematic review, we provided an overview of HRQoL studies that include patients with ACC. We searched Medline, Cochrane library, Google Scholar, and Embase for articles published in English until July 2019. We used the following keywords: adrenocortical carcinoma or adrenocortical neoplasm, Cushing syndrome or hypercortisolism, hyperaldosteronism, androgens/estrogens or virilizing/feminizing, adrenalectomy, radiotherapy, chemotherapy, mitotane, and adrenal insufficiency. We combined these keywords one or two at a time with the search terms quality of life, health status, or well-being. For example, we used the search strategy: (adrenocortical carcinoma or adrenocortical neoplasm) and adrenalectomy and (quality of life or health status or well-being).

We included original articles that contained one or more HRQoL measurements and included patients with ACC. Studies were excluded if the participants were below 18 years of age or if the study only included patients with other adrenal tumors such as benign tumors, metastasis, pheochromocytoma, neuroendocrine tumors, and neuroendocrine carcinomas. Studies that researched animals or cell lines were also excluded. The search, exclusion, and data extraction procedures were performed by two authors independently, and discrepancies were resolved through discussion.

**Figure 1:**
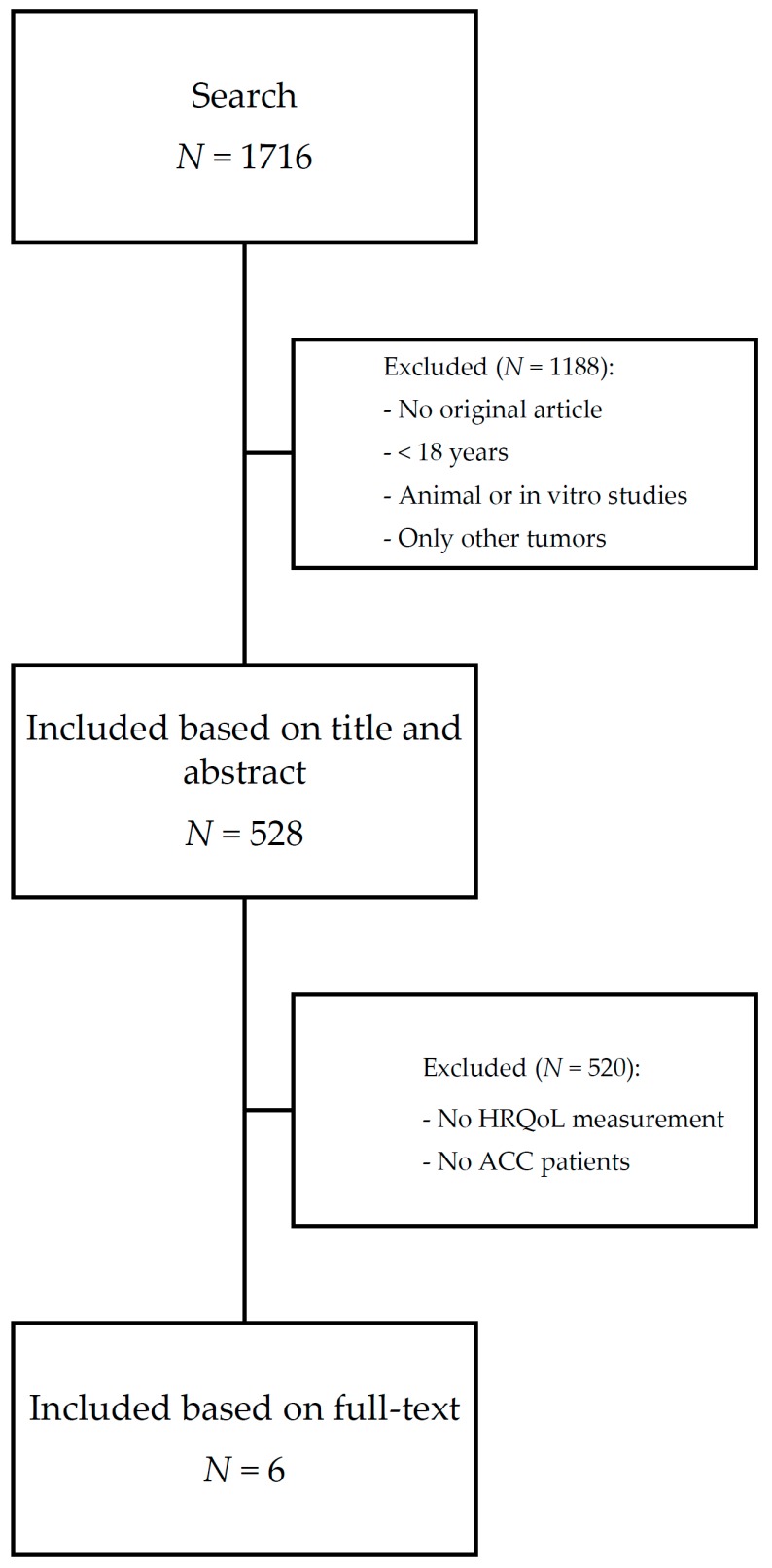
Flow diagram of systematic literature search. ACC: adrenocortical carcinoma; HRQoL: health-related quality of life.

A total of 1716 articles were identified by the search, of these, 528 met the in- and exclusion criteria based on title and abstract as illustrated in Figure 1. After a full-text review, we included a total of six studies that measured HRQoL in 323 patients with ACC, summarized in Table 1.

## 4. Conclusions

Little is known about HRQoL in patients with ACC. There have been six HRQoL studies that included patients with ACC. Four of them included only a few patients with ACC, and did not provide the HRQoL measurements for the patients with ACC separately. One study only provided a positive association between patient satisfaction with care and HRQoL. The only study thus far with an adequate sample of patients with ACC and a validated HRQoL questionnaire is the FIRMACT study that compared two chemotherapy-mitotane regimes [14]. They discovered that the HRQoL of patients with ACC in a palliative setting was reduced compared to the general population. The study also showed that even though one of the regimes had a positive effect on survival, both the treatments had a negative effect on patients’ HRQoL.

Although the number of HRQoL studies in patients with ACC is limited, the literature does indicate with reasons that the course of the disease and the treatments of ACC can have a large impact on patients’ HRQoL. Healthcare professionals can take this potential impact into account when discussing diagnosis and treatment options. For example, patients can experience hypercortisolism, hyperaldosteronism, and adrenocortical insufficiency, which have a large impact on HRQoL in patients with benign diseases as shown in the recent review of Ho and Druce [20]. This impact on HRQoL even persists after recovery of hormonal status. However, we hypothesize that patients with ACC can experience different and additional HRQoL issues due to malignancy of the tumor. Cancer and its treatment has a major impact on patients’ lives. The issues that influence HRQoL in cancer patients are not fully comparable to those in other chronic illnesses [56,58]. These issues have not yet been studied in patients with ACC. Furthermore, the HRQoL effects of mitotane therapy, an important part of the ACC treatment with a high toxicity rate, are still unknown.

### Directions for Further Research

The recent ESE guideline on ACC describes the importance of gathering more information on HRQoL in patients with ACC [2]. In this review we showed that little is known about HRQoL in patients in ACC, and a disease-specific questionnaire does not exist. Knowledge of HRQoL across the course of the disease and treatment options is necessary to understand the impact of the disease on patients’ lives. This knowledge will help communication about HRQoL issues and will aid the shared decision making process [5].

We therefore conclude that there is need for more HRQoL research in patients with ACC to improve patient-centered care. To better aid the processes of shared decision making and treatment guidance, we need more information on the HRQoL impact of all stages of the disease and all treatment options. We therefore recommend that all future trials and cohorts include at least a general HRQoL measurement designed for use in cancer patients. In addition, disease specific HRQoL questionnaires are more sensitive to differentiate between different symptoms of the disease and adverse effects of treatment options. We therefore recommend efforts be taken to develop an ACC-specific HRQoL questionnaire, since patients with ACC can also experience more specific HRQoL issues. The results of this review can be used as a first source of information, in line with the guidelines of questionnaire development set by the EORTC [59].

## Figures and Tables

**Table 1:** Overview of health-related quality of life studies in patients with adrenocortical carcinoma.

Study	Design	Study Population	HRQoL Measure	HRQoL Outcome Overall	HRQoL Outcome ACC
Pikkarainen 1999 [11]	Cross-sectional, surgery (transsphenoidal pituitary or laparotomic adrenalectomy)	Overall: 74 CS ACC: 4	VAS	Improved HRQoL after surgery	Not provided (2 alive)
von Essen 2002 [12]	Cross-sectional	Overall: 85 gastro-intestinal endocrine tumors ACC: 9	EORTC QLQ-C30, HADS, CASC	Patient satisfaction associated with HRQoL and anxiety	Not provided
Sippel 2008 [13]	Cross-sectional, laparoscopic adrenalectomy	Overall: 60 CS ACC: 2	HRQoL improved yes/no	Improved HRQoL after surgery	Not provided (1 alive)
Fassnacht 2012 [14]	FIRMACT, RCT double blind, EPD-mitotane vs streptozotocine-mitotane	Overall: 304 ACC	EORTC QLQ-C30	No difference in HRQoL between regimes before and after treatment, reduced HRQoL compared to general population	Median EORTC QLQ-C30 score: Baseline 58.3/100 (*n* = 204) After treatment 50.0/100 (*n* = 129, *p* for change 0.996)
Fleseriu 2012 ^1^ [15]	Open clinical trial, mifepriston treatment	Overall: 50 CS with DM and/or HT ACC: 3	SF36, BDI	Reduced HRQoL compared to general population, improved HRQoL and reduced depression after treatment	Not provided
Katznelson 2014 ^1^ [16]	Open clinical trial, mifepriston treatment	Overall: 50 CS with DM and/or HT ACC: 3	SF36, BDI	Reduced HRQoL across all components except body pain	Not provided
Dovirak 2016 [17]	Cohort, laparoscopic adrenalectomy	Overall: 30 ACC: 1	SF12, CARE	Reduced HRQoL and physical activity and increased pain after 2 weeks, recovery of HRQoL after 4 weeks	Not provided

ACC: adrenocortical carcinoma; BDI: Beckwith depression index; CARE: convalescence and recovery evaluation; CASC: comprehensive assessment of satisfaction with care; CS: Cushing’s syndrome; DM: diabetes mellitus; EDP: etoposide, doxorubicin, cisplatin; EORTC QLQ-C30: European organization for research and treatment of cancer quality of life questionnaire-C30; HADS: hospital anxiety and depression scale; HT: hypertension; NEC: neuro-endocrine carcinoma; HRQoL: health-related quality of life; RCT: randomized controlled trial; SF-36/12: Short Form 36/12; VAS: visual analogy scale. ^1^ Same research population and measurements.

**Table 2:** Adverse effects of mitotane monotherapy.

Clinical Effects	Laboratory Disturbances
Common—very common	
● Gastrointestinal: nausea, vomiting, diarrhea, anorexia, mucositis ● Central nervous system: lethargy, somnolence, vertigo, ataxia, confusion, depression, dizziness, decreased memory, paresthesia, myasthenia ● Adrenal insufficiency ● Gynecomastia ● Skin rash ● Primary hypogonadism in men	● Increase of hepatic enzymes (γ-GT) ● Increase in hormone binding globulins (CBG, SHBG, TBG, vitamin D binding protein) ● Disturbance of thyroid parameters (interference with binding of T4 to TBG, total T4H ↓) ● Hypercholesterolemia, hypertriglyceridemia ● Hepatic microsomal enzyme induction with increased metabolism of glucocorticoids and other steroids and barbiturates, phenytoin, warfarin ● Leukopenia ● Prolonged bleeding time
Rare—very rare	
● Liver failure ● Autoimmune hepatitis	● Thrombocytopenia, anemia ● Hematuria, albuminuria
Incidence not known	
● Cardiovascular: hypertension ● Ocular: blurred vision, double vision, toxic retinopathy, cataract, macular edema ● Hemorrhagic cystitis	

GT: Glutamyl transpeptidase; CBG: cortisol-binding globulin; TBG: thyroxin-binding globulin. Adapted from Fassnacht, et al. ESE guideline 2018 [2].
